# Influence of inflammation and nitric oxide upon platelet aggregation following deposition of diesel exhaust particles in the airways

**DOI:** 10.1111/bph.13831

**Published:** 2017-05-27

**Authors:** E Smyth, A Solomon, M A Birrell, M J Smallwood, P G Winyard, T D Tetley, M Emerson

**Affiliations:** ^1^ Platelet Biology GroupNational Heart and Lung Institute, Imperial College LondonLondonUK; ^2^ Respiratory PharmacologyNational Heart and Lung Institute, Imperial College LondonLondonUK; ^3^ Inflammation Research GroupUniversity of Exeter Medical SchoolExeterUK; ^4^ Lung Cell Biology GroupNational Heart and Lung Institute, Imperial College LondonLondonUK

## Abstract

**Background and Purpose:**

Exposure to nanoparticulate pollution has been implicated in platelet‐driven thrombotic events such as myocardial infarction. Inflammation and impairment of NO bioavailability have been proposed as potential causative mechanisms. It is unclear, however, whether airways exposure to combustion‐derived nanoparticles such as diesel exhaust particles (DEP) or carbon black (CB) can augment platelet aggregation *in vivo* and the underlying mechanisms remain undefined. We aimed to investigate the effects of acute lung exposure to DEP and CB on platelet activation and the associated role of inflammation and endothelial‐derived NO.

**Experimental Approach:**

DEP and CB were intratracheally instilled into wild‐type (WT) and eNOS^−/−^ mice and platelet aggregation was assessed *in vivo* using an established model of radio‐labelled platelet thromboembolism. The underlying mechanisms were investigated by measuring inflammatory markers, NO metabolites and light transmission aggregometry.

**Key Results:**

Platelet aggregation *in vivo* was significantly enhanced in WT and eNOS^−/−^ mice following acute airways exposure to DEP but not CB. CB exposure, but not DEP, was associated with significant increases in pulmonary neutrophils and IL‐6 levels in the bronchoalveolar lavage fluid and plasma of WT mice. Neither DEP nor CB affected plasma nitrate/nitrite concentration and DEP‐induced human platelet aggregation was inhibited by an NO donor.

**Conclusions and Implications:**

Pulmonary exposure to DEP and subsequent platelet activation may contribute to the reports of increased cardiovascular risk, associated with exposure to airborne pollution, independent of its effects on inflammation or NO bioavailability.

**Abbreviations**
BALF: broncheoavleolar lavage fluid
CB: carbon black
DEP: diesel exhaust particles
eNOS: endothelial NOS
eNOS^−/−^: endothelial NOS knockout mice
MI: myocardial infarction
PM: particulate matter

## Introduction

Exposure to ambient particulate matter (PM) is associated with thrombotic events such as myocardial infarction (MI) and stroke (Peters *et al.,* 2001). The PM 0.1 fraction of air pollution, which includes diesel exhaust particles (DEP) and carbon black (CB), is principally combustion‐generated from vehicle emissions and is strongly implicated as a causative risk factor for these cardiovascular events (Andersen *et al.,* 2010). Platelet activation and aggregation are important drivers of the thrombotic events associated with exposure to PM 0.1 (Nemmar *et al.,* 2003a; Lucking *et al.,* 2008; Solomon *et al.,* 2013).

Lung exposure to DEP has been shown to enhance experimental thrombosis and platelet activation in both animal models (Nemmar *et al.,* 2003a,b) and man (Lucking *et al.,* 2008). Exposure to both DEP and CB has been associated with inflammation (Nemmar *et al.,* 2003a,b; Gilmour *et al.,* 2004; Lucking *et al.,* 2008) and initiation of pulmonary and systemic inflammation has been proposed as a mechanism by which PM 0.1 inhalation may promote platelet aggregation and thrombosis (Oberdorster *et al.,* 1992; Mills *et al.,* 2005). IL‐6 has been particularly associated with diesel exhaust (DE) or DEP exposure in both rodents (Robertson *et al.,* 2012; Conklin, Kong, and Committee, 2015) and humans (Krishnan *et al.,* 2013). In addition, leukocyte infiltration in the lungs is widely reported following exposure to DEP (Oberdorster *et al.,* 1992; Nemmar *et al.,* 2003b; Robertson *et al.,* 2012; Xu *et al.,* 2013). In contrast, DEP was able to accelerate thrombosis in rats without evidence of pulmonary or systemic inflammation (Tabor *et al.,* 2016) so that the role of inflammation in driving DEP‐associated cardiovascular risk remains unclear.

As an alternative to the inflammation mechanism, it has been reported that nanoscale materials can translocate across the pulmonary epithelial barrier and enter the circulation. Thus, nanoparticles may interact directly with blood components including platelets (Nemmar *et al.,* 2001; Oberdorster *et al.,* 2002; Kreyling *et al.,* 2009). Previous work by us showed that DEP can physically interact with isolated platelets leading to activation and aggregation as well as enhancement of aggregation at lower concentrations (Solomon *et al.,* 2013). Furthermore, systemic administration of DEP by the i.v. route, to mimic a scenario in which PM 0.1 had traversed the lung epithelium, caused enhanced platelet aggregation *in vivo* (Solomon *et al.,* 2013) and thrombogenesis (Tabor *et al.,* 2016). Binding of DEP to glycoprotein VI (GPVI) and/or C‐type lectin‐like receptor‐2 (CLEC‐2) has been suggested to be an underlying mechanism behind DEP‐induced platelet aggregation (Alshehri *et al.,* 2015).

The *in vivo* experimental models of thrombosis primarily used to date are vascular injury models and *ex vivo* preparations which involve multifactorial thrombotic processes and do not distinguish specific impacts on platelets. Although DEP administered i.v. has been shown to increase platelet aggregation *in vivo*, the distinct effect on platelet aggregation following administration to the airways remains unclear.

It is reported that DEP can reduce the bioavailability of NO possibly *via* uncoupling of endothelial NOS (eNOS) or increased oxidative stress (Knuckles *et al.,* 2008; Langrish *et al.,* 2013; Wauters *et al.,* 2013). As NO generated in the vascular endothelium is a major endogenous negative regulator of platelet activation (Emerson *et al.,* 1999; Moore *et al.,* 2010; Moore *et al.,* 2011), reduced NO bioavailability following exposure to DEP could hypothetically lead to enhanced platelet activation.

We hypothesized that acute exposure of the mouse airways to DEP or CB would modulate platelet aggregation *in vivo.* We used an established mouse pharmacological model of radiolabelled (with [^111^In]) platelet aggregation to explore our hypothesis and evaluated whether any observed effects on platelet aggregation were associated with pulmonary leukocyte infiltration, IL‐6‐mediated inflammation or related to NO activity.

## Methods

### Preparation of washed human platelets

Informed consent was obtained from all donors and the procedures were approved by the NHS National Research Ethics Service. Washed platelet suspensions were prepared from citrated blood from consenting, healthy, aspirin‐free, male and female human donors (23–55 years) as previously described (Jones *et al.,* 2010).

### Light transmission aggregometry

Human platelets were stimulated with either DEP at concentrations previously shown to induce concentration‐dependent aggregation (12–50 μg·mL^−1^) (Solomon *et al.,* 2013), collagen (5 μg·mL^−1^) or HEPES‐buffered Tyrode's solution (THB; composition; 134 mM NaCl, 2.9 mM KCl, 12 mM NaHCO_3_, 0.34 mM Na_2_HPO_4_, 20 mM HEPES, 10 mM glucose and 1 mM MgCl_2_; pH 7.4) and aggregation was measured in an optical aggregometer (Chrono‐log Corporation, Haventown, PA, USA) for 3 min as previously reported (Solomon *et al.,* 2013). The NO donor sodium nitroprusside (SNP) (10 μM) was incubated with platelets for 5 min prior to agonist stimulation. Having consulted the ARRIVE guidelines (Kilkenny *et al.,* 2010), it was considered unnecessary to conduct *in vitro* experiments in mice on the grounds that it could be reasonably concluded that observations made in humans would apply to mice. Given that the human platelet response is the endpoint of interest, *in vitro* experiments in mice could not be justified from a 3Rs perspective.

### Animals

All animal care and experimental procedures were performed in accordance with our Home Office licence and with authorization from the Imperial College London Ethical Review Panel. Protocols were refined in association with the National Centre for Replacement, Refinement and Reduction of Animals in Research (NC3Rs) (Tymvios *et al.,* 2008) and are reported in accordance with the ARRIVE guidelines for reporting experiments involving animals (Kilkenny *et al.,* 2010; McGrath & Lilley, 2015). Male C57BL/6 mice (Harlan, Bicester, UK) between 20–25 g were used. eNOS knockout mice (eNOS^−/−^, strain 0026847) (18–25 g) were purchased from Jackson Laboratory (Bar Harbour, ME, USA) and bred in‐house. Food and water were available *ab libitum*, and animals were housed in groups of five in individually ventilated cages*.* Animals were randomized to treatment groups. Details of anaesthesia are provided below. At the end of procedures, animals were killed by cervical dislocation.

### Preparation of nanoparticles

DEP and CB particles were prepared in sterile saline (NaCl 0.9%) or THB and sonicated prior to use using techniques previously described and shown to generate nanoparticulate suspensions (Solomon *et al.,* 2013).

### Intratracheal instillation of diesel exhaust particles and carbon black

WT and eNOS^−/−^ mice were anaesthetised with isoflourane (4%), and a 50 μL bolus of either saline (0.9% *w*/*v*), CB or DEP (25 μg/mouse) was administered through a Hamilton syringe® attached to an oral gavage steel feeding tube. Mice were left to recover in a surgical recovery box, for 4 h prior to the experiments.

### Bronchoalveolar lavage and plasma collection and cell counting

Mice were terminally anaesthetised with urethane (10 μL·g^−1^ 25% *w*/*v* i.p), bronchoalveolar lavage fluid (BALF) was collected as previously described (Raemdonck *et al.,* 2016). Total and differential cell counts were performed as previously described (Raemdonck *et al.,* 2016). Citrated blood was collected *via* cardiac puncture from mice terminally anesthetized with urethane (10 μL·g^−1^ 25% *w*/*v* i.p.). Blood was centrifuged at 300 rcf for 3 min and plasma stored at −80°C until further analysis.

### In vivo platelet aggregation

A previous model of *in vivo* platelet aggregation was employed (Tymvios *et al.,* 2008). Citrated blood was collected *via* cardiac puncture from mice (WT or eNOS^−/−^) terminally anesthetized with urethane (2 kg^−1^ i.p.). Platelets were isolated and radiolabelled with [^111^In] as previously described (Tymvios *et al.,* 2008). Terminally anesthetized mice (1.5 g·kg^−1^ urethane i.p.) were i.v. infused with radiolabelled platelets prior to i.v. administration of collagen (50 μg·kg^−1^). Entrapment of platelet aggregates in the pulmonary vasculature following aggregation in the systemic circulation was quantified by recording changes in radioactive counts for 5 min with a Single Point Extended Area Ratio probe (eV products, Saxonburg, PA) positioned over the pulmonary vasculature and custom software (Mumed systems, London, UK) as previously described (Tymvios *et al.,* 2008).

### Histological analysis of nanoparticle pulmonary deposition following intratracheal instillation

Immediately following i.t. instillation mice were killed *via* cervical dislocation and their lungs were dissected, briefly washed in PBS and fixed in formalin overnight. Following this, samples were embedded in wax, sectioned (longitudinally) and stained (haematoxylin and eosin). The samples were viewed using a light microscope (×20 and ×40).

### ELISA

WT mouse cytokines (IL‐6) were quantified in cell free BALF and plasma using a standard sandwich elisa (DuoSet elisa kits, R&D Systems, USA) as per the manufacturer's instructions. All samples were run in triplicate with the appropriate controls and the standard curve was run in duplicate. Cytokine concentrations were calculated using a standard curve.

### Ozone chemiluminescence for measurement of plasma nitrate

Nitrate/nitrite concentrations were measured in plasma from mice treated with DEP or CB (25 μg per mouse) *via* i.t. instillation, using ozone chemiluminescence with a Sievers NO analyser (280; Analytix, Boldon, UK, as previously reported (Apostoli *et al.,* 2014).

### Experimental design, data and statistical analysis

Data and statistical analysis in this study comply with the recommendations on experimental design and analysis in pharmacology (Curtis *et al.,* 2015). For most protocols, blinding was not feasible as experiments were conducted by an individual experimenter; however, for histological analyses, blinding was used. Unless otherwise stated, data is presented as box‐and‐whisker plots; the horizontal lines inside the box indicate the median. The box edges extend from the 25th to the 75th percentiles, and the whiskers represent the minimum and maximum values. Statistical analyses were performed on raw data using Prism 5 Graphpad software. Two‐way analyses were conducted using a Mann–Whitney test and multiple comparisons were conducted using a Kruskal–Wallis test with Dunn's comparison. *Post hoc* tests were run only if there was no significant variance in homogeneity. Power calculations were conducted (at level 0.8) to determine appropriate n numbers. A *P* value <0.05 was indicative of statistical significance.

### Materials

Materials used were supplied as follows: [^111^In]indium oxine (Mallinckrodt Radiopharmacy Services, London, UK); collagen (Nycomed, Munich, Germany); DEP (SRM 2975; National Institute of Standards and Technology, Gaithersburg, MD, USA); CB (Printex‐90; Degussa GmbH, Hanau, Germany); cytokine elisas DuoSet (R&D systems, Minnesota, USA). All other materials were purchased from Sigma‐Aldrich (Poole, UK) and were of analytical grade.

### Nomenclature of targets and ligands

Key protein targets and ligands in this article are hyperlinked to corresponding entries in http://www.guidetopharmacology.org, the common portal for data from the IUPHAR/BPS Guide to PHARMACOLOGY (Southan *et al.,* 2016), and are permanently archived in the Concise Guide to PHARMACOLOGY 2015/16 (Alexander *et al.,* 2015a,b).

## Results

### Diesel exhaust particles and carbon black were deposited in the conducting airways

**Figure 1:**
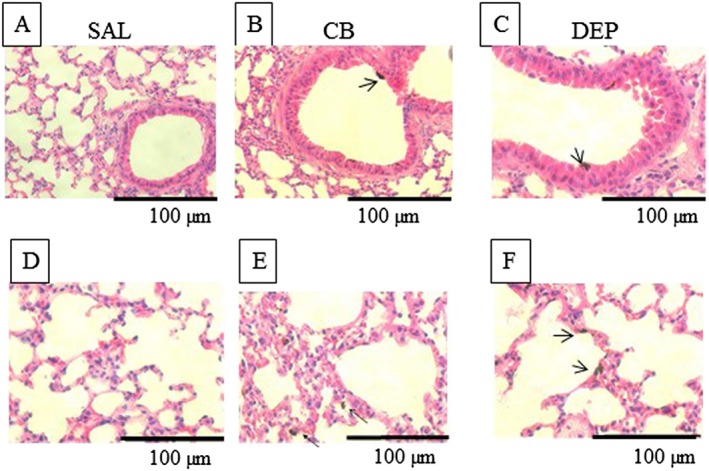
Deposition of DEP and CB in mouse lungs following i.t. instillation. Anaesthetised WT mice were i.t. instilled with DEP (C,F) and CB (B,E; 25 μg per mouse) or saline (SAL) (NaCl 0.9%) (A,D). Mice were killed *via* cervical dislocation immediately after i.t. instillation, and their lungs were removed and fixed in formalin. Samples were embedded, sectioned and stained with haematoxylin and eosin. Slides were visualized on a light microscope at ×20 000 and ×40 000. Sections from five mice exposed to each treatment were observed, and representative images are shown.

Becuase a significant proportion of PM 0.1 inhaled by humans is deposited in the distal airways (ICRP, 1994), the location of DEP (Figure 1C,F) and CB (Figure 1B,E) following i.t. instillation was examined, in mice. The lung microarchitecture was similar between the saline control, DEP and CB exposed airways (Figure 1). Aggregates of CB and DEP were observed in contact with the pulmonary epithelial cells (Figure 1E,F) and conducting airways (Figure 1B,C). These data confirm that i.t. instillation of nanoparticles leads to their deposition deep in the airways.

### Diesel exhaust particle deposition in the airways enhanced platelet aggregation in vivo

**Figure 2:**
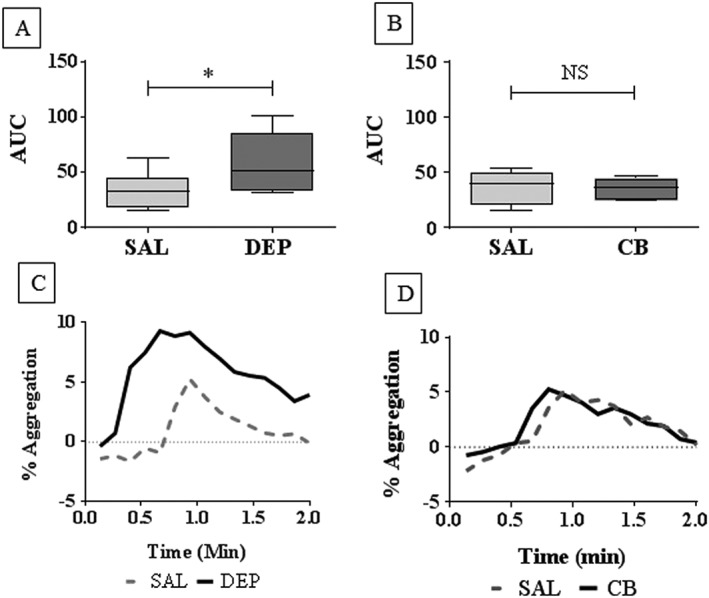
Platelet aggregation *in vivo* following i.t. instillation of DEP, CB or saline (SAL). WT mice were i.t. instilled with DEP, CB (25 μg per mouse) or sterile SAL (0.9%). Isolated platelets were radiolabelled with [^111^In]‐indium oxine and infused into mice 4 h following i.t. instillation, prior to injection with collagen (50 μg·kg^−1^ i.v.). Responses were recorded as changes in counts for 10 min. Platelet aggregation was assessed by changes in percentage maximum increase in scintillation counts (A,B) or AUC (C,D). Data are presented as median ± interquartile range (A,B) or representative traces (C,D), *P < 0.05, significantly different as indicated; Kruskal–Wallis test with Dunn's comparison. NS = non‐significant, *n* = 8 (A) or 6 (B).

Having shown deposition of DEP and CB in the conducting airways, we explored whether this was associated acutely (4 h later) with an enhancement of agonist‐induced platelet aggregation *in vivo* using a model of radiolabelled platelet thromboembolism. There was a significant increase in collagen‐induced platelet aggregation *in vivo* measured as AUC (*P* < 0.05 compared to saline control) 4 h following i.t. administration of DEP (Figure 2A,C). In contrast, there was no significant enhancement of the AUC (Figure 2B,D; *P* > 0.05 compared to saline control) following administration of CB.

### Carbon black exposure induced pulmonary and systemic inflammation

**Figure 3:**
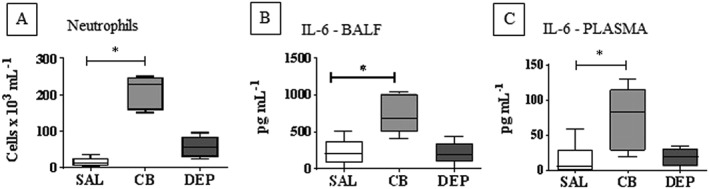
Inflammatory profile in BALF and plasma following i.t. instillation of CB, DEP or saline (SAL). WT mice were i.t. instilled with DEP, CB (25 μg per mouse) or sterile SAL (0.9% NaCl). BALF was obtained 4 h following i.t. instillation *via* catheterisation of the trachea and instillation of 400 μL of sterile SAL (NaCl 0.9%) for 30 s (×3). Blood was obtained 4 h following i.t. instillation *via* cardiac puncture. Neutrophils (A) and IL‐6 (B) levels were measured in the BALF and IL‐6 levels were measured in the plasma (C). Data are presented as median and interquartile range. *P < 0.05, significantly different as indicated; Kruskal–Wallis test with Dunn's comparison, *n* = 5 (A) or 6 (B,C).

Having shown contrasting impacts of DEP and CB upon *in vivo* platelet aggregation, we investigated whether these effects were associated with changes in inflammatory markers. A significant increase in both neutrophils and IL‐6 (*P* < 0.05 compared to saline; Figure 3A,B) was detected in BALF following exposure to CB. In contrast, no significant increases in neutrophils (Figure 3A) or IL‐6 (Figure 3B; *P* > 0.05 compared to saline) were observed in BALF following administration of DEP. Plasma levels of IL‐6 were also significantly increased following administration of CB but not DEP (Figure 3C). Neither DEP nor CB caused a detectable change in other leukocytes in BALF (data not shown). DEP therefore enhanced *in vivo* platelet aggregation in the absence of an effect upon the inflammatory parameters measured whereas CB produced a detectable inflammatory response without impacting platelet aggregation.

### Diesel exhaust particles enhanced platelet aggregation in the absence of eNOS

**Figure 4:**
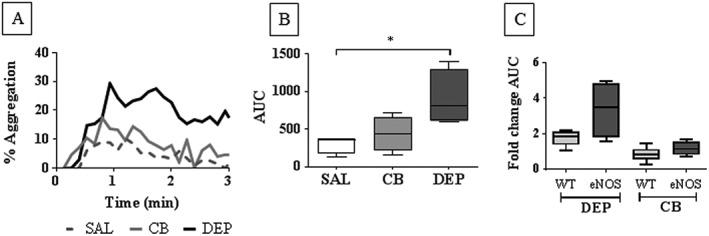
Platelet aggregation *in vivo* in eNOS^−/−^ mice following i.t. instillation of DEP, CB or saline (SAL). eNOS^−/−^ mice were i.t. instilled with DEP, CB (25 μg per mouse) or sterile SAL (0.9%). Isolated platelets were radiolabelled with [^111^In]‐indium oxine and infused into mice 4 h following i.t. instillation, prior to injection with collagen (50 μg·kg^−1^). Responses were recorded for 10 min as changes in counts over time. Platelet aggregation was assessed by changes in percentage increase in scintillation counts (A) or AUC (B). Fold change from the SAL control in percentage maximum increase in scintillation counts (C) following i.t. instillation of DEP and CB in WT and eNOS^−/−^ mice. Data are presented as median ± interquartile range (B,C) or representative traces (A). *P < 0.05, significantly different as indicated; Kruskal–Wallis test with Dunn's comparison or Mann–Whitney signed rank test, *n* = 6 (A) or 5 (B).

We explored the hypothesis that DEP exerted effects *via* eNOS by employing eNOS^−/−^ mice. A lack of effect of DEP upon platelet aggregation in eNOS^−/−^ would confirm this hypothesis. Following i.t. instillation of DEP into eNOS^−/−^ mice, a significant enhancement of the AUC (*P* < 0.05 compared to saline control) in response to collagen was observed after 4 h (Figure 4A,B). No significant changes in AUC were detected for CB exposed eNOS ^−/−^ mice (Figure 4A,B). Thus, the effect of DEP upon platelet aggregation *in vivo* is not dependent upon the presence of eNOS.

We also analysed the fold‐increase in AUC following DEP or CB exposure relative to saline responses in order to determine whether platelet responses were proportionally different in eNOS^−/−^ compared to WT. Although no significant differences were observed, there was a non‐significant trend towards an increased proportional response following DEP exposure in eNOS^−/−^ *versus* WT (Figure 4C).

### DEP and NO‐mediated regulation of platelets

**Figure 5:**
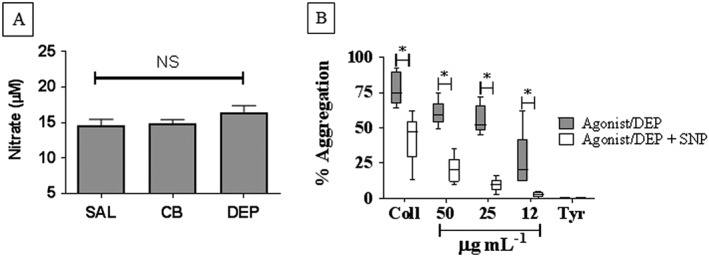
The effects of carbonaceous nanoparticles on NO bioavailability and effects of NO on DEP‐induced platelet aggregation Mice were i.t. instilled with DEP, CB (25 μg per mouse) or sterile saline (SAL, 0.9%). Blood was taken *via* cardiac puncture, 4–5 h following exposure. Nitrate and nitrite levels were measured in plasma using gas‐phase chemiluminescence (nitrite levels were below the detection limit of the assay (50 nM) and therefore data are presented as nitrate concentrations) (A). Isolated human platelets were exposed to DEP (12–50 μg·mL^−1^) or collagen (Coll; 5 μg·mL^−1^) following a 5 min incubation with either a NO donor sodium nitroprusside (SNP; 10 μM) or Tyrode's buffer (Tyr; B). Changes in light transmission were measured for 3 min. Data are presented as box‐and‐whisker plots (A) and as median ± interquartile range (B), NS = non‐significant compared using Kruskal–Wallis test with Dunn's comparison (A), *P < 0.05, significantly different from time matched controls using Wilcoxon signed rank test (B). *n* = 6 (A) or 5 (B).

Neither DEP nor CB instillation caused any significant alteration in plasma nitrate levels (Figure 5A) indicating a lack of a detectable effect on NO bioavailability. Nitrite levels were below the detection limit (50 nM) of the assay. Having previously shown that DEP can induce platelet aggregation (Solomon *et al.,* 2013) as well as a trend towards greater aggregation in the absence of eNOS (Figure 4C), we investigated whether DEP‐induced platelet aggregation was sensitive to the negative regulator of platelet activation, NO. Isolated human platelet aggregation induced by collagen or DEP (12–50 μg·mL^−1^) was significantly inhibited by SNP (10 μM) (*P* < 0.05; Figure 5B). Typical raw data traces showing the effect of SNP on collagen‐ and DEP‐induced aggregation are provided as a Supporting Information Figure. Thus, we did not find evidence that the ability of DEP to enhance platelet aggregation was driven by changes in NO bioavailability. Under our experimental conditions *in vivo*, loss of NO did not significantly enhance aggregation. However, our *in vitro* data do not exclude the possibility that NO may inhibit the ability of DEP to directly induce platelet aggregation should it translocate across the epithelial barrier.

## Discussion and conclusions

In this study, we explored the hypothesis that introduction of DEP into the airways could lead to an enhancement of subsequent platelet aggregation in an acute time frame. One of the principal reasons why inhaled PM 0.1, such as DEP, is considered to be responsible for the harmful effects of ambient PM is its deposition within the lower airways including the alveolar gas exchange units (ICRP, 1994). In the present study, agglomerates of DEP and CB were observed both in the conducting airways and the distal alveoli confirming appropriate lung penetration following i.t. administration. I.t. instillation therefore, to some extent, mimics inhalation of nanoaparticles into the airways. It is not, however, possible from the histological analyses conducted to observe individual nanoparticles nor to assess their translocation across the lung epithelium. It is important to address the relevance of the doses of nanoparticles employed in this study with reference to human exposure. More than 20 mg of particulates can be inhaled by humans in polluted cities every 24 h (Nemmar *et al.,* 2003a). Our dosing of 25 μg equates to a total burden of 2 mg·kg^−1^ body weight so although our study was not intended to mimic human exposure, the levels used are probably relevant if one considers an exposure period of a few days.

The thrombotic events associated with inhalation of PM 0.1 are known to involve platelet activation and aggregation but, currently, there is limited information regarding the influence of DEP and CB on platelet function *in vivo*. The present study showed that DEP, but not CB, administered to the lungs of mice *via* the instillation route caused a significant increase in agonist‐induced platelet aggregation *in vivo* 4 h post‐exposure. These results indicate that the thrombotic events associated with acute pulmonary exposure to PM 0.1 may be driven mechanistically by platelets.

Supportive to the present study, acute exposure to DEP by i.t. instillation has been demonstrated to enhance thrombosis and platelet activation in rodent models after 30, 60 (Nemmar *et al.,* 2003a) or 120 min (Tabor *et al.,* 2016). Similarly, thrombosis was enhanced *in vivo* following a 24 h exposure of lungs to DEP (Nemmar *et al.,* 2009). Although there are limited clinical studies, increased thrombosis has been reported in human subjects using *ex vivo* perfusion techniques following an acute inhalation exposure (2–4 h) to DE (Lucking *et al.,* 2008). In the current study, DEP has been shown to affect platelet aggregation independent of the other components of the thrombotic response as the model employed has been shown to be driven entirely by platelet aggregation *in vivo* (Tymvios *et al.,* 2008) and in the presence of an intact endothelium (Tymvios *et al.,* 2008; Emerson, 2010) suggesting that DEP may affect platelets in the absence of endothelial dysfunction or injury.

Inflammation is associated with increased cardiovascular risk (Mameli *et al.,* 2009). One hypothesis regarding how PM 0.1 may promote thrombosis is therefore by induction of pulmonary inflammation, spill‐over of inflammatory cytokines into the systemic circulation and consequential platelet activation (Seaton *et al.,* 1995). In particular, exposure to PM has been linked with thrombotic events driven by IL‐6 (Mutlu *et al.,* 2007) and leukocyte influx to the airways (Nemmar *et al.,* 2003a; Nemmar *et al.,* 2009). No increases in neutrophils or IL‐6 were observed following DEP exposure in the current study. There was therefore no evidence of an inflammatory response to DEP in our model as no measured hallmarks of pulmonary inflammation were detected. This finding conflicts with some published data in which DEP has been reported to cause increases in pulmonary neutrophils 1 and 6 h following pulmonary delivery in hamsters (Nemmar *et al.,* 2003a) and rats (Robertson *et al.,* 2012). In contrast, other reports support our findings, demonstrating dissociation of the thrombogenic effects of DEP from pulmonary and systemic inflammation (Tabor *et al.,* 2016). Differences in the inflammatory impacts of DEP reported in various studies may be due in part to differences in species studied. Regardless of this, it is apparent that enhanced platelet activation following exposure to DEP can occur independently of quantifiable changes in the inflammatory response in the context of the parameters assessed in the current study.

In terms of human studies, acute exposure to DE caused changes in thrombosis formation but no alterations in systemic cytokines or acute phase proteins (Lucking *et al.,* 2011). Additionally, no changes in systemic inflammatory markers were detected in healthy men and men with coronary artery disease exposed to DEP for 1 h with intermittent exercise (Mills *et al.,* 2005; Mills *et al.,* 2007) or 2 h after exposure to concentrated ambient particles (Mills *et al.,* 2008). However, increases in systemic IL‐6 and TNF‐α 24 h following exposure to DEP have been reported in humans (Tornqvist *et al.,* 2007), animals (Robertson *et al.,* 2012) and at longer >24 h time intervals (Nemmar and Inuwa, 2008). Based on the current data, it appears that DEP may not necessarily induce systemic inflammation following acute exposure and therefore may not be the underlying mechanism behind the enhanced platelet aggregation observed in the present work. The possibility that inflammation may drive platelet activation in other models and at different exposure periods cannot be excluded. Since nanoparticles have been suggested to translocate the lung to enter the blood (Kreyling *et al.,* 2009) and DEP can directly enhance platelet activation platelets following physical interaction (Solomon *et al.,* 2013), an alternative or additional hypothesized mechanism is translocation of DEP across the lung epithelium and subsequent exposure and activation of circulating platelets. The ultimate objective in proving this hypothesis would be visualization of DEP in contact with activating platelets following i.t. administration or inhalation of DEP, but this has not yet been demonstrated and is technically challenging.

In contrast to DEP, administration of CB to mice caused significant increases in neutrophils and IL‐6 in the BALF and IL‐6 in the plasma. Systemic inflammation has similarly been reported following a single 7 h exposure of rats to unfractionated CB (Gilmour *et al.,* 2004). The release of pro‐inflammatory cytokines by CB further supports the conclusion that systemic inflammation was not the primary underlying mechanism behind the DEP‐induced enhanced platelet response, as CB did not alter platelet aggregation *in vivo* but was associated with systemic inflammation. Thus, PM 0.1‐associated inflammation was not linked to platelet aggregation in our study. The contrasting effects of DEP and CB may suggest the constituents present on the surface of DEP to be responsible for the effects on platelet aggregation since the presence of these surface compounds and metals distinguishes the two pollutants. Further work to identify the biological effects of these surface components of DEP will provide valuable information on the mechanisms underlying the health impact of combustion derived air pollutants.

A hypothesized mechanism driving DEP‐enhanced platelet aggregation that was explored in this study was that DEP can reduce the bioavailability of endogenous NO and thereby reduce the inhibitory influence NO has on platelets. Acute exposure to DE has been reported to induce endothelial dysfunction *via* uncoupling of eNOS (Knuckles *et al.,* 2008). DEP has also been demonstrated to enhance vasoconstriction, potentially due to reduced NO bioavailability (Langrish *et al.,* 2013). If reduced NO bioavailability were responsible for the effects of DEP on platelet aggregation then DEP would be expected to have no effect on platelet aggregation in eNOS^−/−^ mice. Since DEP caused an increase in platelet aggregation in eNOS^−/−^ mice and did not appear to influence plasma nitrate and nitrite, there was no evidence of reduced NO bioavailability as an underlying mechanism driving increased platelet aggregation. Since NO negatively regulates platelet activation, we explored whether loss of eNOS in fact led to enhanced platelet aggregation. DEP had no significant effect on platelet aggregation in eNOS^−/−^ relative to in WT so that an effect of endogenous NO upon DEP‐enhanced platelet aggregation could not be inferred. To explore whether, mechanistically, DEP‐induced platelet aggregation could be impacted by NO, we conducted studies *in vitro*. DEP‐induced platelet aggregation was significantly inhibited by a NO donor. The extent of inhibition observed was similar to that achieved with collagen and is in keeping with the proposed GPVI‐mediated mechanism by which DEP is proposed to activate platelets (Alshehri *et al.,* 2015). The finding of an inhibitory effect of NO in regulating DEP‐mediated platelet aggregation *in vitro*, although not supported by the *in vivo* studies in this paper, is sufficient to hypothesize that when platelets are directly exposed to DEP, potentially following translocation across the lung epithelium, NO may act to oppose platelet activation. This suggestion is in keeping with epidemiological data that demonstrates the greatest cardiovascular risk in individuals with risk factors associated with endothelial dysfunction (Brook *et al.,* 2002; Mills *et al.,* 2005; Brauner *et al.,* 2008; Wauters *et al.,* 2013). Further investigation is warranted to ascertain whether NO acts in a physiologically cardioprotective manner to reduce DEP‐driven cardiovascular risk.

In conclusion, acute airways exposure to DEP can enhance platelet aggregation responses *in vivo* and this may provide a mechanism for the thrombotic effects that are associated with acute exposure to PM. Additionally, the mechanisms underlying this enhanced platelet response do not appear to involve the initiation of systemic inflammation or alterations in NO bioavailability. Our study does, however, generate a hypothesis that healthy individuals may be protected from the harmful cardiovascular effects of DEP that comes into direct contact with platelets, through the inhibition of DEP‐induced platelet activation by NO.

## Author contributions

E.S.: design, conduct and analysis of experiments and drafting of manuscript; A.S., M.A.B. and M.J.S.: design and conduct and analysis of experiments; P.G.W.: concept and design of experiments; T.D.T.: concept and design of experiments and drafting of manuscript and revising for critically important intellectual content; M.E.: concept and design of experiments, drafting of manuscript and revising for critically important intellectual content and final approval of manuscript.

## Conflict of interest

The authors declare no conflicts of interest.

## Declaration of transparency and scientific rigour

This Declaration acknowledges that this paper adheres to the principles for transparent reporting and scientific rigour of preclinical research recommended by funding agencies, publishers and other organisations engaged with supporting research.

## Supporting information


**Figure S1** Original, unmodified typical *in vitro* isolated platelet aggregation traces showing changes in light transmission (A) in response to collagen (5 μg mL^‐1^) following a 5 min incubation with either Tyrode's buffer (Coll) or the NO donor sodium nitroprusside (SNP, 10 μM). (B‐C) Response to diesel exhaust particles (DEP) following Tyrode's or SNP at (B) 25 μg mL^−1^ or (C) 50 μg μl^−1^. Typical traces of *n* = 5 are shown.
