# Supplementary material for: Influence of inflammation and nitric oxide upon platelet aggregation following deposition of diesel exhaust particles in the airways
Source: Br J Pharmacol. 2017 May 27;174(13):2130–9. doi: 10.1111/bph.13831 (PMC5466527; doi:10.1111/bph.13831)
Supplement: Supplementary file 1 — Figure S1 Original, unmodified typical in vitro isolated platelet aggregation traces showing changes in light transmission (A) in response to collagen (5 μg mL‐1) following a 5 min incubation with either Tyrode's buffer (Coll) or the NO donor sodium nitroprusside (SNP, 10 μM). (B‐C) Response to diesel exhaust particles (DEP) following Tyrode's or SNP at (B) 25 μg mL−1 or (C) 50 μg μl−1. Typical traces of n = 5 are shown. [file BPH-174-2130-s001.pdf]

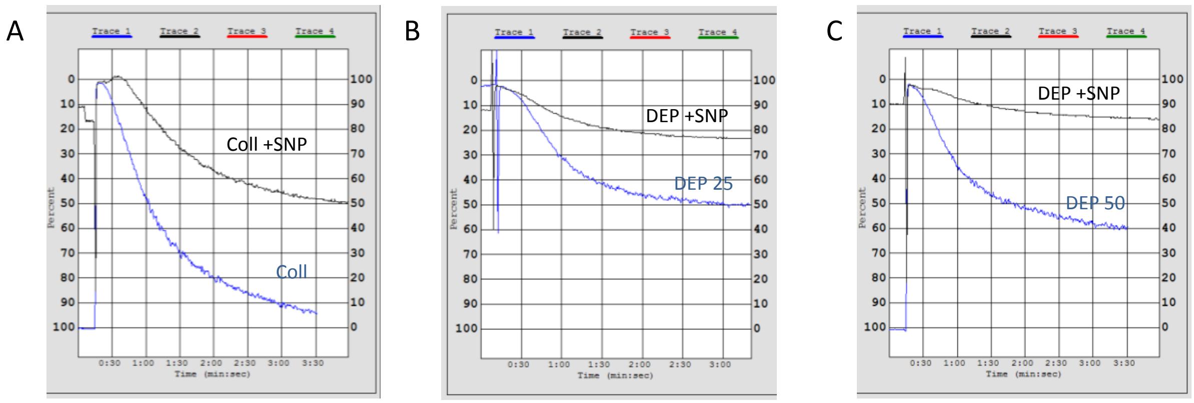

Supplementary Fig. Original, unmodified typical *in vitro* isolated platelet aggregation traces showing changes in light transmission (A) in response to collagen (5 µg/ml) following a 5 min incubation with either Tyrode's buffer (Coll) or the NO donor sodium nitroprusside (SNP, 10 µM). (B-C) Response to diesel exhaust particles (DEP) following Tyrode's or SNP at (B) 25 µg ml^{-1} or (C) 50 µg μl^{-1}. Typical traces of n=5 are shown.
